# The Development of a Postgraduate Orthopaedic Manual Therapy Residency Program in Nairobi, Kenya

**DOI:** 10.3389/fpubh.2017.00153

**Published:** 2017-06-30

**Authors:** Shala Cunningham, Richard Jackson, Daniel Kangutu Muli, Joni McFelea

**Affiliations:** ^1^Department of Physical Therapy, Radford University, Roanoke, VA, United States; ^2^The Jackson Clinics Foundation, Inc., Middleburg, VA, United States; ^3^Department of Physiotherapy, Kenya Medical Training College, Nairobi, Kenya; ^4^Department of Physical Therapy, University of Evansville, Evansville, IN, United States

**Keywords:** Residency program, Kenya, clinical reasoning, physiotherapy, manual therapy

## Abstract

**Introduction:**

There are very few opportunities for long-term, comprehensive postgraduate education in developing countries because of fiscal and human resource constraints. Therefore, physiotherapists have little opportunity following graduation to advance their skills through the improvement of clinical reasoning and treatment planning and application.

**Background:**

To address the need for sustainable advanced instruction in physiotherapy within the country, a postgraduate Residency program was initiated in Nairobi, Kenya in 2012. The mission of the program is to graduate advanced orthopedic practitioners who can lead their communities and local profession in the advancement of clinical care and education. Since its inception, six cohorts have been initiated for a total of 90 resident participants. In addition, six program graduates are being trained to continue the Residency program and are serving as teaching assistants for the on campus modules. This training will result in a self-sustaining program by 2020.

**Discussion:**

The manual therapy Residency education model allowed for advancement of the participating physiotherapists professional development utilizing evidence-based practice. This was done without altering the current education system within the country, or accessing expensive equipment.

**Concluding remarks:**

The Residency program was developed and established with the cooperation of a local education institution and a non-profit corporation in the United States. This collaboration has facilitated the advancement of orthopedic clinical standards in the country and will, hopefully, one day serve an as a template for future programs.

## Introduction

In the year 2000, it was estimated that there were 234 million moderately or severely disabled people living in developing countries (1). This number is projected to grow to 525 million in 2035 (1). Although there are limited numbers of physiotherapists available to provide services in these countries, there is a possibility to maximize the potential and skills of the physiotherapists who do exist for the benefit of those in need of services. However, there are very few opportunities for long-term, comprehensive postgraduate clinical education in developing countries because of fiscal and human resource constraints. Therefore, physiotherapists have little opportunity to improve clinical reasoning and treatment skills (2). To promote the profession of physiotherapy in Kenya, an orthopaedic Residency program was developed in Nairobi, Kenya.

## Background

Physiotherapists in Kenya currently have the opportunity to earn a 3-year diploma or a Bachelor of Science degree in the field of physiotherapy (3). According to the World Confederation for Physical Therapy, education for entry-level therapists should include a minimum of 4 years of university level courses (4). In addition, physiotherapists should be committed to pursuing educational opportunities following entry-level education to promote the development of the profession (4). Access to advanced instruction, fundamental to promoting educational development, is limited in Kenya. One factor restricting advanced instruction has been the shortage of physiotherapists with advanced degrees and specialty training to offer educational opportunities following entry-level education (2).

### Development of the Residency Program

To assist with the progression of clinical reasoning and skill development, an Orthopaedic Manual Therapy Residency (Residency) program was introduced in Nairobi, Kenya in 2012 (5). The Residency is a partnership between the Jackson Clinics Foundation (Foundation) in the United States (US) and Kenya Medical Training College (KMTC) in Nairobi. The Foundation is a non-governmental organization formed for the purpose of funding humanitarian efforts in Africa (5). The Foundation provides the recruitment and transportation of qualified instructors from universities and clinics throughout the US to Nairobi, Kenya. KMTC secures housing for instructors, provides teaching space for the program, and grants a Higher Diploma in Orthopaedic Manual Therapy to successful graduates of the Residency program. The mission of the Higher Diploma Program is to graduate advanced orthopedic practitioners who can lead their communities and local profession in the advancement of clinical care and education.

Multiple steps were taken to establish a long-term educational program, including comprehensive didactic education and clinical mentoring, to improve clinical practice and health-care delivery by physiotherapists in Kenya. During the development of the program, meetings were held with key stakeholders; the Foundation, the director of the KMTC, and the head of the department of physiotherapy education. Discussions centered on a shared vision and mission for the program. Common goals for the program were agreed upon. In addition, details regarding what each stakeholder could provide to ensure the success of the program were examined. Following the development of the mission for the program and drafting of a Memorandum of Understanding, the goals and scope of the program were shared with the University of Nairobi and Kenyatta University to ensure that misunderstandings did not ensue. In addition, the program was presented to the Ministry of Health. Once the program was accepted by all parties, the development of the content was addressed by the Foundation and physiotherapy faculty at KMTC.

### Program Content

The physiotherapists participating in the Orthopaedic Manual Therapy Residency program have a 3-year technical diploma in physiotherapy and have reported no previous access to continuing education throughout their careers. The residents complete six modules over 18 months. The online didactic portion of the program utilizes the Clinical Practice Guidelines and Current Concepts in Orthopedics, 3rd edition (American Physical Therapy Association) as background reading and preparation for participation in onsite modules (6). Each module takes place during 10 days of onsite education and mentoring provided by physical therapy instructors from the US. Instructor qualifications include currently a faculty member teaching in the area of orthopedics within an accredited physical therapy program or having an advanced certification in both orthopedics and manual therapy. The purpose of each module is to provide the residents with the didactic education and clinical skills consistent with the orthopedic curriculum provided by professional doctorate in physical therapy programs in the US.

In addition to onsite modules and online resources, residents receive clinical mentoring focused on integrating the knowledge and skills learned during the Residency program into clinical practice. Although 90% of residents work within the Nairobi area, 10% work outside of the city. To allow for mentoring of these residents, KMTC contracts with providers to allow the residents to practice as students within local facilities. To progress in the program, residents must achieve adequate performance on written and practical examinations provided at the completion of each module. Following completion of the 18-month Residency program, residents must successfully pass a comprehensive written examination and a live patient practical examination to earn the Higher Diploma.

### Participants

Since 2012, 51 volunteers from the US have provided instruction in the Residency program as didactic instructors and clinical mentors to the Residents. The first cohort of the program graduated in December 2014, and the second cohort graduated in December 2015. Currently, four additional cohorts are in progress of completing the Residency program for a total of 90 Residents. Although significant support has been provided through the Foundation to institute the program, a train-the-trainer program has been established. Six graduates are currently being trained to continue the Residency program and are serving as teaching assistants for the on campus modules. Mentoring is being provided to the teaching assistants to allow progression of their understanding of the content and effective delivery skills for teaching both didactic and procedural knowledge. The training of graduates to provide ongoing education will result in a self-sustaining program by 2020.

### Costs

The estimated cost of the Residency program over 6 years is $600,000. The Foundation has provided the costs associated with airfare from the US to Kenya for volunteers. Furthermore, the Foundation has sought professionals with specialty training in manual therapy to provide mentoring for the residents in their current clinical setting focused. This mentoring focuses on integrating the knowledge and skills learned during the Residency program into clinical practice. KMTC provides the resources and facilities needed for the onsite modules. These include a large gym space with standard treatment tables and exercise equipment and audiovisual equipment. KMTC also arranges lodging and transportation for the volunteer instructors and mentors. Residents pay $1,100 in tuition for the program (the equivalent of three months salary) to offset some of the local costs. As a gift from the Foundation to successful graduates, a messenger bag with an inclinometer, two goniometers, pinwheel, reflex hammer, exercise band, stethoscope, pulse oximeter, and blood pressure cuff is provided at graduation. This provides the graduates with some tools for to providing treatment to patients.

## Discussion

Even though residents verbally relayed their gratitude for the education, a formal program evaluation was initiated to begin to explore the outcomes of the Residency program and discover any facilitators and/or barriers for participation in the program. In addition, the Foundation hoped to determine obstacles to the integration the newly acquired knowledge and skills gained through the Residency into clinical practice. Outcomes were explored through a mixed methods approach with interviews and a paper survey.

### Outcomes

All 15 residents in the first (2012) cohort of the Residency program consented to participate in the outcome measurement process.

#### Interviews

Individual interviews with residents were conducted following completion of their final live patient practical examination. Interviews were audio recorded and transcribed by an independent transcriptionist to ensure accuracy. The descriptive phenomenology approach was utilized to describe the data. The information from the interviews was coded using the constant comparative method by two investigators, and general themes were identified specifically addressing facilitators and barriers for participation in the Residency program and the implementation of new skills in clinical practice. Thick descriptions and narrative of the participants were provided to inform the themes. The following themes were identified: (1) there was an initial discontent expressed by colleagues regarding participation in the Residency and the required time away from the clinic to participate in onsite modules; (2) patients’ required education regarding manual therapy treatment techniques to be comfortable with the new approach to care; (3) residents experienced a decrease in productivity as they were practicing new skills and assessment techniques in the clinic; (4) there was a positive impact on the ability to utilize clinical reasoning to determine multiple hypotheses for the patient’s diagnosis and then effectively determine a working diagnosis for which to choose evidence-based treatment techniques; and (5) mentoring in the clinic was the most effective method for integrating knowledge into practice.

#### Survey

The primary researcher developed a 19-item written survey to determine the impact of the Residency program on professional development and career advancement. The survey was adapted from a tool that had been used previously in studies related to outcomes of residency training in the US (7, 8). The survey contained 12 questions related to professional development. Each question had six possible responses: “major positive,” “some positive,” “no effect,” “some negative,” “major negative,” and “unable to assess.” Cronbach’s alpha for the 12 questions related to professional development was 0.884. The survey also contained seven questions related to the influence of the Residency program on career advancement. Because the alpha for the seven items calculated at 0.672, two items related to career fulfillment and research opportunities were removed from the questionnaire based on this assessment. The responses two items were consistent for all 15 residents. Alpha for the five remaining questions related to career advancement was 0.713. Similar to the results found in the US (7, 8), the graduates reported a positive influence of the Residency training on their professional development including their ability to: perform a thorough clinical examination; use a logical clinical reasoning process; determine the nature of a patient’s problem; treat complex patients; treat effectively to achieve projected outcomes; perform overall patient management; use scientific literature to provide rational for interventions; critically read and evaluate scientific literature; communicate with patients; and communicate with other health professionals. In addition, graduates reported an increase in the number of patient referrals and the number of professionals referring patients to them. However, in regard to career advancement; the residents reported no change in salary, job promotion, or access to research opportunities related to participation in the program.

## Concluding Remarks

Residency programs emphasizing clinical reasoning and manual therapy could provide a means to optimize the effects of physiotherapy (minimize pain, normalize movement, and maximize function) without the need for or access to expensive equipment. The Residency format provided in Kenya could allow physiotherapists with technical degrees access to specialty training and ongoing mentorship without a substantial change to educational infrastructure for entry-level education currently provided at higher education institutions within developing countries. The outcomes from the first cohort of the Residency program and the continued positive collaboration between institutions in the US and Kenya suggest that the program is positioned for a sustainable impact on patient care. The success of this program may provide a template for the future development of similar long-term programs in countries with limited resources.

## Ethics Statement

This research was approved by the Kenya Medical Training College Ethics and Research Committee and the University of Evansville Institutional Review Board. Residents were made aware of the upcoming study by the residency coordinator in Kenya through verbal communication in June 2014. In September 2014, the primary investigator traveled to Kenya. She discussed the purpose of the study, procedures associated with the study (survey completion and one-on-one interviews), and requirements for time involvement up to 50 min. Written consent was obtained. Instructors in the Residency program were not aware of which residents consented to participate in the study, and participation did not affect the residents performance or standing within the program.

## Author Contributions

SC: developed the methodology for surveys and qualitative research performed to investigate outcomes associated with the Residency program; analyzed the results of the quantitative and qualitative data. RJ: instrumental in developing the curriculum for the Residency program in Kenya; collaborated with the Kenya Media Training College for develop and initiate the Residency program; and assisted with writing information regarding the development of the program. DM: instrumental in coordinating efforts with administrators from both the United States and Kenya; coordinated data collection in Kenya. JM: assisted with methodology development for the outcomes research associated with the Residency program.

## Conflict of Interest Statement

RJ is the founder of the Jackson Clinics Foundation. The remaining authors declare that the research was conducted in the absence of any commercial or financial relationships that could be construed as a potential conflict of interest.
